# Refractive error characteristics and influence on ocular parameters in patients with unilateral congenital ptosis

**DOI:** 10.1186/s12886-022-02511-x

**Published:** 2022-07-02

**Authors:** Yingli Liu, Tingting Chen, Jingwen Huang, Wentao Li, Yilin Chen, Lijun Huo

**Affiliations:** Department of Ophthalmology, First Affiliated Hospital of Sun Yat-sen University, 58 Zhongshan 2nd Road, Yuexiu District, Guangzhou, Guangdong Province 510080 P. R. China

**Keywords:** Congenital ptosis, Refractive error, Ocular parameters, IOL master700

## Abstract

**Background:**

The study aimed to investigate the difference in refractive status and ocular parameters between ptotic and fellow eyes in patients with unilateral congenital ptosis.

**Methods:**

Thirty patients (53% males, age 22.00 ± 11.41 years) with unilateral congenital ptosis diagnosed and treated at the First Affiliated Hospital of Sun-yat Sen University were enrolled and underwent detailed refractive examinations from March 2019 to February 2022. Ocular biometric measurements were performed by an IOL Master 700 biometer. The differences in refractive error characteristics, best-corrected visual acuity (BCVA), and ocular parameters including axial length (AL), central corneal thickness (CCT), aqueous depth (AQD), anterior chamber depth (ACD), lens thickness (LT), and keratometry values between ptotic and fellow eyes were analysed.

**Results:**

A lower BCVA (logMAR, median (IQR), 0.00 (− 0.13,0.00), *P* = 0.009) and a higher incidence of amblyopia (n (%), 7(23%), *P* = 0.016) were observed in ptotic eyes. The CCT of ptotic eyes was greater than that of fellow eyes (mean ± SD, 539.83 ± 26.73 μm, *P* < 0.001). The keratometry values at the flat axis (K1) and mean corneal power (Km) were smaller in ptotic eyes (mean ± SD, 42.11 ± 1.49 D, 42.68 ± 1.52 D, respectively, both *P* = 0.001). There was no significant difference in AL between ptotic and fellow eyes.

**Conclusions:**

Congenital ptosis influences ocular parameters, mainly causing a thicker and flatter cornea. Patients with unilateral congenital ptosis might have lower BCVA in the ptotic eyes.

**Supplementary Information:**

The online version contains supplementary material available at 10.1186/s12886-022-02511-x.

## Introduction

Congenital ptosis is a relatively normal ocular abnormality caused by a drooping eyelid within the first year of life, characterized as a narrow palpebral fissure and more coverage by the upper eyelid on the cornea. Several studies have reported that congenital ptosis can increase the incidence of myopia, hyperopia, astigmatism, anisometropia and amblyopia [1–5]. Previous studies have shown that pressure from the upper eyelid may alter corneal shape [6]. In animal experiments, axial elongation was induced by covering the pupil was observed [7–9]. This effect was also illustrated in human infants [10]. However, under long-term pressure and coverage from the upper eyelid, the effect of congenital ptosis on ocular structure remains unclear.

Recently, the IOL Master 700 (Carl Zeiss AG, Jena, Germany), a highly advanced, noncontact biometer, was introduced, allowing accurate ocular parameter testing on patients with congenital ptosis [11]. It is based on swept-source optical coherence tomography (SS-OCT), providing fixation points for the patients and retinal OCT images for technicians when testing to gain accurate axial measurements from the posterior cornea to the fovea [12]. In addition, it also provides measurements of central corneal thickness, lens thickness, anterior chamber depth, and corneal power.

In this study, we focus on both refractive status and the ocular biological parameters of unilateral congenital ptosis to compare the refractive error between ptotic eyes and fellow eyes and to investigate the influence of congenital ptosis on the ocular structure during eyeball growth.

## Materials and methods

### Patients

We enrolled 30 patients with unilateral congenital ptosis who were diagnosed and treated at the Ophthalmology Department of the First Affiliated Hospital of Sun Yat-sen University between March 2019 and February 2022. This study was approved by the Ethics Committee of the First Affiliated Hospital of Sun Yat-sen University in China ([2021]544) and complied with the tenets of the Declaration of Helsinki for biomedical research involving human subjects.

The normal position of the upper lid covers the cornea by less than 2 mm, and ptosis can be diagnosed when the upper lid is below its normal position. The severity of ptosis can be classified as mild (1–2 mm), when the upper lid does not cover the pupil, moderate (3–4 mm), when the upper lid partially covers the pupil, and severe (more than 4 mm), when the pupil is completely covered by the eyelid [13].

The inclusion criteria included unilateral congenital ptosis, age greater than 5 years, absence of other ocular pathology, and reliable measurement results. The exclusion criteria were acquired ptosis, patients younger than 5 years and inability to cooperate with the examination, a history of eye trauma, previous ocular surgery (including ptosis correction surgery), corneal opacity, wearing of an eye contact lens in the last 3 months, and other ophthalmic or systemic disorders.

### Ophthalmic examination

All patients were examined by the same trained examiner. Routine ophthalmic examinations were performed for all patients, including best-corrected visual acuity (BCVA) measurements using a Snellen chart, detailed slit-lamp examination of the anterior segment, fundus examination with dilated pupils, and measurement of intraocular pressure (Topcon CT-80A, Tokyo, Japan). Complete evaluation of ptosis, including measurement of the height of the palpebral fissure (HPF) and levator functions (LF), was performed by the lid excursion method, involving measurement of the upper lid from extreme downgaze to extreme upgaze with the frontalis muscle fixed.

### Refractive error examination

Cycloplegic refraction was performed for all patients. Children younger than 10 years were given 1% atropine ointment once a day for 3 days and were examined on the fourth day. Patients older than 10 years were examined following administration of combined 0.5% phenylephrine hydrochloride and 0.5% tropicamide eye drops three times every 5 minutes. We calculated the refraction by the spherical equivalent refraction (SER) and defined myopia as an SER of at least − 0.50 D and hyperopia as an SER of + 2.00 D or more. Astigmatism was defined as 0.75 DC or more. Anisometropia was defined as at least a 1.00 D difference with the opposite eye. We defined amblyopia as a visual acuity of 6/9 or less and a difference of more than two Snellen lines between the fellow and ptotic eyes, excluding any other ocular abnormalities [1, 14].

### Ocular biometric measurements

Biometric measurements were performed using an IOL Master 700 three times by the same well-trained technician before pupillary dilatation in all patients. Each time before the examination, the device was recalibrated. During the measurement, the examinees were asked to position their heads on a headrest and look at a fixation point for foveal scans. Poor-quality results were deleted, and new measurements were obtained until the readings were reliable.

The axial length (AL), central corneal thickness (CCT), aqueous depth (AQD), anterior chamber depth (ACD), lens thickness (LT), keratometry values at the flat (K1) and steep (K2) axis, and mean corneal power (Km) were obtained. AL was defined as the distance from the tear film to the retinal pigment epithelium (RPE) of the fovea. CCT was defined as the distance from the anterior to the posterior cornea [15]. AQD was measured using signals from the posterior face of the cornea to the anterior surface of the lens. The ACD was defined as the distance from the central corneal epithelium to the anterior surface of the lens and calculated by adding the CCT and AQD (CCT + AQD) [16]. LT was defined as the distance between the anterior and posterior lens surfaces. Corneal power included K1, K2 and Km, where K1 was defined as the corneal power at the flat axis and K2 at the steep axis, and Km was the average value of K1 and K2 [17, 18].

### Statistical analysis

Statistical analysis was performed by using SPSS version 22.0. The Snellen VA was converted to the logarithm of the minimum angle of resolution (logMAR) value. The Shapiro–Wilk test was used to examine whether the measured values followed a normal distribution. Descriptive results for continuous variables are expressed as the mean ± standard deviation (SD) or median and interquartile range (IQR) depending on the normality of the distribution. Differences were compared using paired t tests for data with a normal distribution and Wilcoxon rank-sum tests for those with a nonnormal distribution. McNemar’s tests were used to compare the frequencies of refractive types between the fellow and ptotic eyes. A *P* value less than 0.05 was considered statistically significant.

## Results

In total, 30 eyes of 30 patients with unilateral congenital ptosis in this study were analysed, and the 30 fellow eyes without ptosis served as controls. The distribution of sex was 16 (53%) males and 14 (47%) females. The average age was 22.00 ± 11.41 years, ranging from 5 to 46 years old. Out of 30 patients, 8 (27%) had mild ptosis, 15 (50%) had moderate ptosis, and 7 (23%) had severe ptosis. Fifteen (50%) had the right eye affected, while 15 (50%) had the left eye affected. Table 1 shows the demographics of the patients.

**Table 1:** Demographic characteristics of the participants (*N* = 30)

Characteristic	
**Age (year, mean ± SD)**	22.00 ± 11.41
**Sex, n (%)**	
Male	16 (53%)
Female	14 (47%)
**Side of affected eye n (%)**	
Right	15 (50%)
Left	15 (50%)
**Severity of ptosis, n (%)**	
Mild	8 (27%)
Moderate	15 (50%)
Severe	7 (23%)

*Abbreviation*: *SD* Standard deviation

The BCVA of the ptotic eyes was significantly lower (logMAR, median (IQR), 0.00 (−0.13, 0.00), *p* = 0.009) than that of the fellow eyes. The incidence of myopia and astigmatism did not differ significantly between ptotic eyes and fellow eyes. However, the prevalence of amblyopia in the ptotic eyes (n (%), 7(23%), *p* = 0.016) was higher than that in the fellow eyes (n (%), 0). All 7 amblyopic eyes were ptotic, 3 severe and 4 moderate. The results are shown in Table 2.

**Table 2:** Differences in BCVA and refractive status between fellow and ptotic eyes (*N* = 30)

	Fellow eye	Ptotic eye	*P* value
**BCVA (logMAR, median (IQR))**	0.00(−0.04, 0.00)	0.00(− 0.13, 0.00)	0.009*
**DS (D), median (IQR)**	−1.25(−3.81, 0.50)	−0.75(−4.13, 0.31)	0.251
**DC (D), median (IQR)**	−0.50(− 0.82, − 0.25)	−0.50(− 1.25, − 0.43)	0.146
**SER (D), median (IQR)**	−1.50(− 3.94, 0.25)	−1.25(− 4.75, 0.25)	0.546
**Refractive type (number, %)**			
Myopia	21 (70%)	19 (63%)	0.625
Hyperopia	0	1 (3%)	1
Astigmatism	10 (33%)	15 (50%)	0.302
Amblyopia	0	7 (23%)	0.016**

*Abbreviation*: *logMAR* Logarithm of the minimum angle of resolution, *IQR* Interquartile range, *BCVA* Best-corrected visual acuity, *DS* Dioptre of spherical power, *DC* Dioptre of cylindrical power, *SER* Spherical equivalent refraction

* Comparison between ptotic and fellow eyes by Wilcoxon rank-sum tests, *P* < 0.05 indicates statistical significance

** Comparison between ptotic and fellow eyes by McNemar’s tests, *P* < 0.05 indicates statistical significance

Ocular biometric parameters in eyes with ptosis were compared with those of the fellow normal eyes and are summarized in Table 3. The CCT was greater in the ptotic eyes than in the fellow eyes (539.83 ± 26.73 μm vs. 530.03 ± 27.43 μm, *p* < 0.001). The K1 (42.11 ± 1.49 D vs. 42.52 ± 1.41 D, *p* = 0.001) and SE (42.68 ± 1.52 D vs. 43.02 ± 1.41 D, *p* = 0.001) of ptotic eyes were lower than those of fellow eyes, which means that the corneas of the former were thicker and flatter. The parameters AL, AQD, ACD, LT, K2 and IOP were not significantly different between the ptotic and fellow eyes.

**Table 3:** Comparison of ocular biometric parameters between ptotic and fellow eyes (*N* = 30)

	Fellow eyes	Ptotic eyes	*P* value
IOP (mmHg), median (IQR)	16.00 (14.75, 17.00)	16.00 (14.00, 17.00)	0.317
AL (mm), median (IQR)	24.02 (23.28, 25.14)	23.83 (23.18, 25.42)	0.360
CCT (μm), mean ± SD	530.03 ± 27.43	539.83 ± 26.73	<0.001*
AQD (mm), mean ± SD	2.90 ± 0.31	2.89 ± 0.28	0.562
ACD (mm), mean ± SD	3.43 ± 0.31	3.44 ± 0.29	0.883
LT (mm), median (IQR)	3.74 (3.55, 3.85)	3.72 (3.53, 3.81)	0.437
K1 (D), mean ± SD	42.52 ± 1.41	42.11 ± 1.49	0.001*
K2 (D), mean ± SD	43.54 ± 1.48	43.29 ± 1.72	0.078
Km (D), mean ± SD	43.02 ± 1.41	42.68 ± 1.52	0.001*

*Abbreviation*: *IQR* Interquartile range, *SD* Standard deviation, *IOP* Intraocular pressure, *AL* Axial length, *CCT* Central corneal thickness, *AQD* Aqueous depth, *ACD* Anterior chamber depth, *LT* Lens thickness, *K1* Keratometry value at the flat axis, *K2* Keratometry value at the steep axis, *Km* Mean corneal power

*Comparison between ptotic and fellow eyes by Paired t test, *P* < 0.05 indicates statistical significance

## Discussion

It has been established that congenital ptosis may increase the incidence of refractive errors, including myopia, hyperopia, and astigmatism, as well as the incidence of amblyopia and anisometropia [1, 19, 20]. In addition, due to amblyopia or deprivation caused by a drooping eyelid covering the pupil, congenital ptosis may have a negative influence on visual acuity [21]. Our study revealed that patients with congenital ptosis are more likely to experience visual impairment. Among the 30 patients with unilateral ptosis, the BCVA in ptotic eyes was lower than that in fellow normal eyes, which indicated that congenital ptosis may lead to visual defects.

In the present study, the incidence of amblyopia in patients with congenital ptosis was 23%, similar to previous reports [21–23]. In addition, all amblyopic eyes in this study were ptotic, revealing that in patients with unilateral congenital ptosis, amblyopia was found more often in ptotic eyes. In regard to the causes of amblyopia in patients with congenital ptosis, astigmatism accounted for the largest proportion. Ugurbas et al. observed that among patients with congenital ptosis, ptotic eyes had more asymmetric and irregular corneas, resulting in an increased magnitude of astigmatism, which may be associated with a higher incidence of amblyopia [3]. The reports by Huo et al. and Paik et al. independently found that in patients with congenital ptosis, ptotic eyes had a higher frequency of astigmatism and amblyopia [1, 23]. In our study, the 7 amblyopic eyes were ptotic, which may to some extent be related to astigmatism; however, larger samples may be needed to verify this assumption.

In normal human eyes, the upper eyelid covers 1–2 mm of the cornea, while the lower eyelid covers none of it. A previous study showed that the superior cornea is thicker than the inferior cornea [24]. Under the coverage and pressure of the upper eyelid, the superior cornea is chronically hypoxic. This finding can be explained by research showing that compared with the central and inferior corneas, the superior cornea demands more oxygen after lifting the upper eyelid [25, 26]. Consequently, we presumed that the chronic hypoxia induced by a constantly drooped eyelid may cause chronic corneal oedema, leading to thickening of the cornea. In the current study, a thicker central cornea was found in ptotic eyes, which was consistent with the findings of Li X et al. [27] We speculated that in congenital ptosis, ptotic eyes are covered more by the drooping eyelids and are subject to mechanical stress, resulting in chronic hypoxia of the cornea and thus leading to a thicker cornea.

In our study, among the 30 patients with unilateral congenital ptosis, no difference in axial length between ptotic eyes and fellow normal eyes was observed, which is consistent with the reports individually by von Noorden et al. and Takahashi et al. [28, 29] One possible explanation is that by adjusting the head posture, patients with congenital ptosis are able to offset the covering of the upper eyelid, thus avoiding occlusion when looking. This indicates that in human eyes, ocular axial elongation does not arise from a similar mechanism to that in animal models [7–9].

A previous study showed that the pressure from the upper eyelids may influence the shape of the cornea, causing it to steepen [30]. In another study, the authors stated that at primary gaze, the wider the horizontal palpebral fissure was, the flatter the cornea [31]. However, in the current study, we found that both the horizontal corneal power and mean corneal power of ptotic eyes were lower than those of fellow eyes, which indicates that congenital ptosis, with a narrower palpebral fissure and under more pressure from the upper eyelid, may lead to flattening of the cornea.

Several limitations of this research need to be considered. First, the average age of these patients (22.00 ± 11.41 years) is relatively high, since people at these ages in China typically have myopia. It is possible that different results regarding the refractive error and axial length could be obtained from younger patients. The second limitation is that the number of patients included in this study was small despite our having calculated the minimum required sample size in advance. Future research needs to be done to determine additional abnormalities induced by congenital ptosis, providing more useful information for treatment. Third, the ocular parameters in this study seem to be limited, and more parameter testing and more accurate techniques should be applied for patients with congenital ptosis. Finally, in order to better control the study findings, only patients with unilateral congenital ptosis were enrolled, and ocular changes caused by acquired ptosis remain unknown; therefore, further studies are needed.

In summary, we confirmed that congenital ptosis may not only have an influence on refractive error and increase the prevalence of amblyopia but also cause the cornea to become thicker and flatter. The impact of congenital ptosis on axial length seems to be minimal in humans.

## Supplementary Information

**Additional file 1.**

## Data Availability

The datasets used and/or analysed during the current study are available from the corresponding author on reasonable request.

## Abbreviations

SS-OCT: Swept-source optical coherence tomography
BCVA: Best-corrected visual acuity
HPF: Height of the palpebral fissure
LF: Levator function
logMAR: Logarithm of the minimum angle of resolution
SER: Spherical equivalent refraction
DS: Dioptre of spherical power
DC: Dioptre of cylindrical power
AL: Axial length
CCT: Central corneal thickness
AQD: Aqueous depth
ACD: Anterior chamber depth
LT: Lens thickness
K1: Keratometry values at the flat axis
K2: Keratometry values at the steep axis
Km: Mean corneal power
RPE: Retinal pigment epithelium
